# Transmissible topological edge states based on Su–Schrieffer–Heeger photonic crystals with defect cavities

**DOI:** 10.1515/nanoph-2023-0744

**Published:** 2024-01-23

**Authors:** Qiuchen Yan, Rui Ma, Qinghong Lyu, Xiaoyong Hu, Qihuang Gong

**Affiliations:** State Key Laboratory for Mesoscopic Physics & Department of Physics, Collaborative Innovation Center of Quantum Matter & Frontiers Science Center for Nano-optoelectronics, Peking University, Beijing 100871, China; Peking University Yangtze Delta Institute of Optoelectronics, Nantong, Jiangsu 226010, China; Collaborative Innovation Center of Extreme Optics, Shanxi University, Taiyuan, Shanxi 030006, China; Hefei National Laboratory, Hefei 230088, China

**Keywords:** topological photonics, photonic crystal, wavelength division multiplexing, defect cavity, transmissible

## Abstract

Topological photonic crystals have great potential in the application of on-chip integrated optical communication devices. Here, we successfully construct the on-chip transmissible topological edge states using one-dimensional Su–Schrieffer–Heeger (SSH) photonic crystals with defect cavities on silicon-on-insulator slab. Different coupling strengths between the lateral modes and diagonal modes in photonic crystal defect cavities are used to construct the SSH model. Furthermore, two photonic SSH-cavity configurations, called α and β configurations, are designed to demonstrate the topological edge states. Leveraging the capabilities of photonic crystal transverse electric modes with on-chip transmission, we introduced a waveguide to excite a boundary defect cavity and found that the transmission peak of light, corresponding to the topological edge state, can be received in another boundary defect cavity, which is caused by the tunnel effect. Moreover, the position of this peak experiences a blue shift as the defect cavity size increases. Therefore, by tuning the size of the SSH defect cavity, on-chip wavelength division multiplexing function can be achieved, which is demonstrated in experiments. The ultrafast response time of one operation can be less than 20 fs. This work harmonizes the simplicity of one-dimensional SSH model with the transmissibility of two-dimensional photonic crystals, realizing transmissible on-chip zero-dimensional topological edge states. Since transmission peaks are highly sensitive to defect cavity size, this configuration can also serve as a wavelength sensor and a reconfigurable optical device, which is of substantial practical value to on-chip applications of topological photonics.

## 1 Introduction

Photonic crystals (PCs) are materials with a periodic refractive index structure, and their unique optical properties enable them to effectively control the propagation and storage of light [1]–[3]. Among them, microcavities in PCs are miniature optical cavities with high-quality factor. Typically, photons are confined within a very small space, where they undergo multiple reflections, thus forming high-quality factor resonant modes [4]–[6]. These resonant modes can be utilized in the manufacturing of efficient light sources, lasers, optical modulators, sensors, and other optical devices [7]–[12] The performance of PC cavities is influenced by various factors, such as the lattice type of the PCs, the geometry of the cavity, and the refractive index of the materials. In order to achieve specific performance characteristics, precise design and fabrication are required to control these factors in general. In recent years, with the advancement of nanofabrication technologies, producing high-quality PC cavities has become easy and feasible [13]–[17] This has opened up more possibilities for the manufacturing and application of optical devices. Furthermore, with the development of topological physics, researchers have discovered that topological edge states (TESs) based on protected band structures exhibit robustness and resistance to backscattering [18]–[21] Since PCs inherently possess band structures, they serve as an excellent platform for studying topological photonics [22]–[25] As a result, researchers have started to integrate the advantages of topology and PCs to design and manufacture high-performance and robust optical devices. Currently, achieved devices include wavelength-division multiplexer (WDM) [26], [27] based on the TESs in two-dimensional (2D) PCs [10], [28], and acoustic devices with tunable pathways realized by doping trivial PC materials [29]. Though 2D topological PCs exhibit the characteristics of unidirectional transportation and backscattering resistance, their fabrication is complex and requires large bulk size to ensure band structures. In contrast, one-dimensional (1D) topological structures have a simple fabrication process and can be ultracompact [30], [31], offering advantages for integrated optical devices. Till now, by combining the Su–Schrieffer–Heeger (SSH) topological configuration with PC cavities [32], topological PC cavities have also seen rapid development, including robust topological nanolasers at interface domain walls [33], higher-order topological corner states [34], topological lasers implemented using L_3_ PC cavities [35], and optical third-order nonlinear effects [36]. However, based on the zero-dimensional (0D) localized TESs, where the wavevector direction of the boundary modes is perpendicular to the plane, these applications pose challenges for on-chip integration of photonic devices, requiring heterogeneous integration or the use of three-dimensional micro/nano fabrication techniques, significantly increasing the devices’ complexity. On the contrary, if the boundary modes achieved through SSH configurations can transmit within the structural plane, they can be easily employed in the next stages of optical computing.

In order to broaden the scope of photonic topology applications within on-chip integrated photonic circuits, we propose the use of SSH cavities for the generation of transmissible topological edge states. Due to the transmissive capability of transverse electric (TE) modes in PCs, the TE modes are chosen as our research foundation. By employing two coupling methods, lateral and diagonal coupling, we establish TESs based on SSH model by inducing coupling strength differences between two cavity modes. Furthermore, by exciting boundary defect cavity through input waveguide, we observe that light signals can be received at the output waveguide of the opposite boundary defect cavity. There are transmission peaks from the light signals, corresponding to the TESs, and the peak position shifts blue as the defect cavity size increases. Therefore, by tuning the size of the SSH defect cavity, on-chip wavelength division multiplexing function can be achieved, which is demonstrated in experiments. Moreover, the ultrafast response time of one operation can be less than 20 fs due to the tunnel effect, which is unattainable by traditional WDM devices. This work combines the simplicity of 0D TESs with the transmissibility in two-dimensional PCs, resulting in the achievement of on-chip, transmissible, and straightforward 0D TESs. Because the transmission peaks are highly sensitive to defect cavity size, this configuration can also function as a wavelength sensor and a reconfigurable optical device, providing significant practical value for on-chip applications of topological photonics.

## 2 Photonic crystal defect cavities

**Figure 1:**
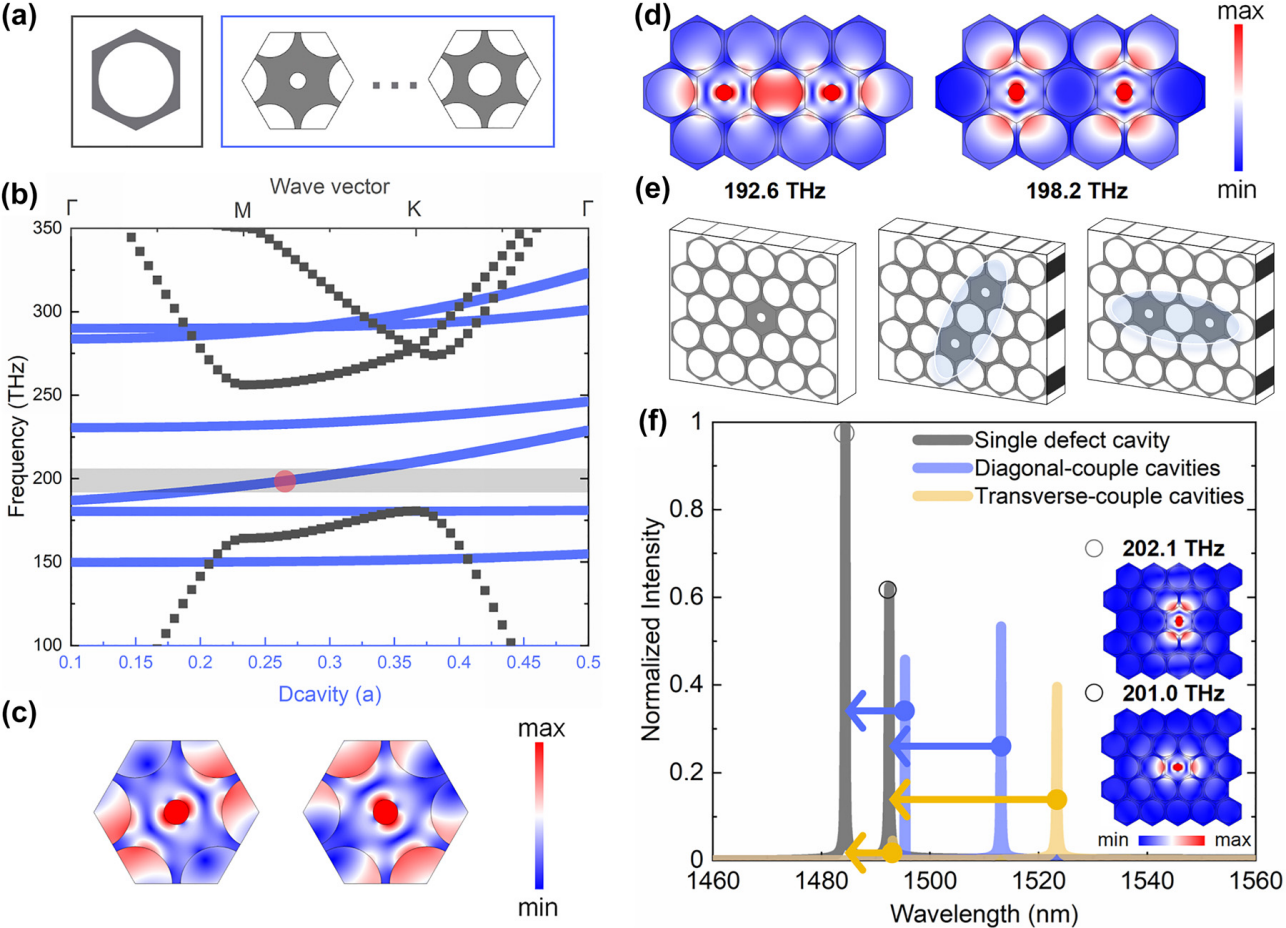
Analysis on the band structures and modes splitting of defect cavities in PC units. (a) Diagrams on trivial PC unit cell and the varied-diameter defect cavities. (b) Band structures of air-hole photonic crystals with the diameter of 0.9*a, represented in grey line. The eigenvalue distributions of varied-diameter defect cavities from the diameter of 0.1*a to 0.5*a, represented in blue line. The light grey region is an optional optical communication band. The red circle represents the resonant frequency of 194 THz for defect cavity with diameter coefficient *d* of 0.27. (c) The two degenerate mode distributions for the point represented by red circle in (b). (d) The mode distributions for a dual-cavity arrangement in which two defect cavities are joined together with a regular PC cavity in between. The lateral mode is at the resonant frequency of 192.6 THz, and the diagonal mode is at the resonant frequency of 198.2 THz. The diameter of defect cavity is 0.3*a. (e) Schematic diagrams of a single defect cavity in a PC unit, two defect cavities in a same-sized PC unit using diagonal coupling arrangement, and two defect cavities in a same-sized PC unit using lateral coupling arrangement. The light blue regions represent coupling units in different coupling arrangements. (f) Mode splitting spectra for single defect-cavity PC unit (grey line), diagonal-coupling arrangement defect-cavity PC unit (blue line), and lateral-coupling arrangement defect-cavity PC unit (yellow line). The inset mode distributions represent the non-degenerate modes in single defect-cavity PC unit, including diagonal mode at resonant frequency of 202.1 THz and lateral mode at resonant frequency of 201.0 THz.

PC cavities have resonant modes that can be classified into TE and transverse magnetic (TM) modes divided by their electric polarization directions. TE modes have electric polarization components parallel to the plane and are typically associated with the air-hole structures in the PCs. TM modes, on the contrary, have electric polarization components normal to the plane and are often associated with the pillar structures in PCs. In PC cavities, TE and TM modes generally exhibit different frequency and energy density distributions. To realize efficient optical devices, it is important to select and control TE and TM modes according to specific application requirements. For square-lattice PCs, composed of a series of dielectric pillars (or air holes) arranged in a square pattern, the band structures of the dielectric pillars with TM mode and the air holes with TE mode can be calculated. In the case of TE modes, there is no photonic bandgap in the square-lattice PCs, making it unable to support TESs. On the contrary, in the case of TM modes, the photonic bandgap appears. Knowing that having bandgaps is an essential condition for generating TESs, however, unfortunately, the use of TM modes will hinder photonic integration. For triangular-lattice PCs, a similar method can be applied to calculate the band structures of the dielectric pillar’s TM mode and the air hole’s TE mode. Both the band structures show photonic bandgaps. In order to further achieve on-chip PC waveguides, we subsequently selected air-holes structures based on triangular lattice as the fundamental research unit, using the TE modes for our study. In this basic unit cell, with a lattice constant, represented by *a*, of 600 nm and the diameter of PC unit cell being 0.9*a, as shown in Figure 1(a), the resulting PC band structure is depicted by the gray line in Figure 1(b).

Subsequently, we replaced the regular PC unit cell in triangular lattice with a defect cavity and examined the influence of the defect cavity’s diameter on the resonant wavelength. The diameter of the defect cavity is represented by the product of the coefficient *d* and the lattice constant *a*. The eigenvalue distributions of varied-diameter defect cavities are as shown by the blue line in Figure 1(b), and the structures diagraming on varied-diameter defect cavities can refer to Figure 1(a). By varying the coefficient *d* from 0.1 to 0.5, there are two degenerate modes in the bandgap. Selecting a specific coefficient *d* of 0.27 with resonant frequency of 194 THz, represented by the red circle in Figure 1(b), there are also some differences in the mode distribution, as illustrated in Figure 1(c). It is worth noting that both of the degenerate modes are excited within the bandgap due to the presence of the defect cavity. In contrast, for trivial PCs without defect cavity, scanning the diameter of all unit cavities according to the coefficient *d* will not yield any usable modes within the bandgap, as shown in Figure SM1.

**Figure 2:**
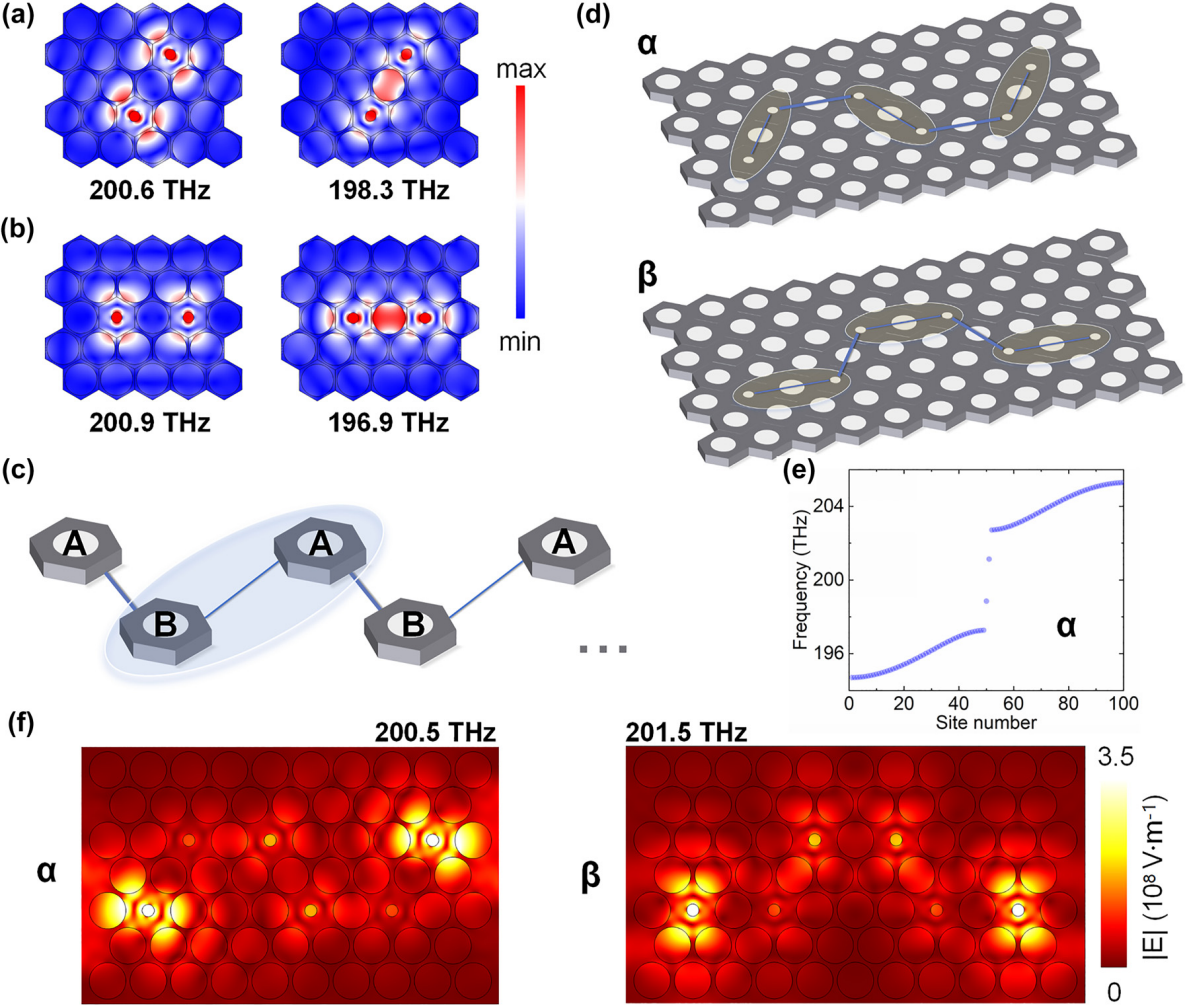
The establishment of TESs with SSH topological model based on coupling strength varying between diagonal modes and lateral modes. (a) The diagonal mode distribution (200.6 THz) and lateral mode distribution (198.3 THz) in the case of defect cavities in a diagonal-couple arrangement PC unit. (b) The diagonal mode distribution (200.9 THz) and lateral mode distribution (196.9 THz) in the case of defect cavities in a lateral-couple arrangement PC unit. (c) Diagram of SSH model, the intra-unit coupling is represented by the thin blue lines, and the inter-unit coupling is represented by the thick blue lines. The circled blue area means a unit including atom A and atom B. (d) Diagrams about the α and β configuration based on SSH model. The α configuration takes the diagonal coupling arrangement as a unit cell, where the circled areas represent the units. The β configuration takes the lateral coupling arrangement as a unit cell, and the units are circled by the light-yellow region. (e) The energy eigenvalue distributions for α configuration, the two isolated solutions in the bandgap correspond to the TESs, and other solutions correspond to bulk states. (f) The simulated TESs distributions of SSH model composed of 6 defect cavities. In the case of α configuration, the TES appears at the frequency of 200.5 THz, and in the case of β configuration, the TES appears at the frequency of 201.5 THz.

Furthermore, in order to study the coupling properties of cavities, two defect cavities can be combined into one unit. Here, we present a dual-cavity arrangement in which two defect cavities are joined together with a regular PC unit cell in between. The diameter of defect cavity is 0.3*a, and its eigenmode distributions are shown in Figure 1(d). The degenerate single defect cavity modes split into two non-degenerate modes, one is at 192.6 THz, referred to as the lateral mode, and the other is at 198.2 THz, known as the diagonal mode. To further investigate the differences in coupling between two defect cavity modes, we first placed a single defect cavity inside a PC unit and then placed two defect cavities inside a same-sized PC unit using both the lateral and diagonal coupling arrangements. Schematic diagrams of the three structures are shown in Figure 1(e). Subsequently, by exciting these structures using dipoles in COMSOL software, we obtained the spectra, as illustrated in Figure 1(f). The gray lines represent the spectrum of the single defect-cavity PC unit, with the resonant frequencies of 202.1 THz, called as diagonal mode, and the resonant frequencies of 201.0 THz, called as lateral mode. The blue lines in Figure 1(f) represent the spectrum of the defect cavities in the diagonal-couple arrangement PC unit, where the diagonal mode has a resonant frequency of 200.6 THz, and the lateral mode has a resonant frequency of 198.3 THz, shown in Figure 2(a). Compared to the modes of single defect cavity, the diagonal mode in the diagonal-couple arrangement exhibits a frequency shift of 1.5 THz, while the lateral mode shows a frequency shift of 2.7 THz. Similarly, the yellow lines in Figure 1(f) represent the spectrum of the defect cavities in the lateral-couple arrangement PC unit. In this case, the diagonal mode has a resonant frequency of 200.9 THz, and the lateral mode has a resonant frequency of 196.9 THz, as seen in Figure 2(b). Compared to the modes of single defect cavity, the diagonal mode in the lateral arrangement displays a frequency shift of 1.2 THz, while the lateral mode exhibits a frequency shift of 4.1 THz. For photonic crystals, the offset in mode splitting can be used to qualitatively reflect the coupling strength, and this can also be explained using a two-level system. Therefore, based on the differences in frequency offsets of diagonal and lateral coupling mode, it is evident that, regardless of the coupling arrangements, the coupling strength of diagonal mode in the dual-defect cavity PC unit is less than that of the lateral mode.

## 3 SSH configurations and topological edge states

TESs are gradually being used to create robust optical devices due to their protection by the band structures. In existing topological models, the SSH model is a classic 1D topological model constructed with chiral symmetry-protected TESs. The diagram of the SSH model is shown in Figure 2(c), where the two PC cavities, represented by A and B, enclosed in the blue region constitute a unit. The coupling strength within the unit is called intra-unit coupling strength, represented by thin blue lines, while the coupling strength between units is called inter-unit coupling strength, represented by thick blue lines. In the SSH model, TESs can be generated when the inter-coupling strength is greater than the intra-coupling strength. According to the results from Section 2, the coupling strength of the dual-cavity diagonal mode is less than that of the lateral mode. Therefore, the diagonal mode in the dual-cavity PC unit can be considered as intra-unit coupling mode, and the lateral coupling mode as inter-unit coupling mode. Since the dual-cavity coupling arrangements are further divided into diagonal and lateral coupling arrangement, two defect-cavity SSH configurations can be constructed based on different coupling arrangements. As shown in Figure 2(d), the α configuration takes the diagonal coupling arrangement of dual defect cavities as a unit cell, where the circled areas represent the unit structures. The β configuration takes the lateral coupling arrangement of dual defect cavities as a unit cell, as shown in the circled areas in the figure. Therefore, the Hamiltonian for the α configuration can be written as Eq. (1) shows.

(1) $$\begin{array}{ll} \hat{H} & =\kappa_{dm}^{da}\overset{n}{\underset{m=1}{\sum }}\left(\left|m,B><m,A\right|+h\cdot c\cdot \right)\\ & \;+\kappa_{lm}^{la}\overset{n-1}{\underset{m=1}{\sum }}\left(\left|m+1,A><m,B\right|+h.c.\right) \end{array}$$

where $\kappa_{dm}^{da}$ means the coupling strength of diagonal mode under diagonal arrangement, and $\kappa_{lm}^{la}$ means the coupling strength of lateral mode under lateral arrangement. *A* and *B* represents different atoms, respectively. Moreover, *m* means the site number and *n* means the total number of sites, also known as the external degrees of freedom in the SSH model. The first term represents the intra-unit coupling process, and the second term represents the inter-unit coupling process. Fix the external degree of freedom of 100, representing the SSH model composed of 100 defect cavities, and then solve for the energy eigenvalues of the Hamiltonian, the eigenvalue distribution is shown in Figure 2(e). There are two isolated points within bandgap, representing TESs with odd and even symmetry [37]–[41]. These two kinds of TESs mode distributions are demonstrated in supplementary material (SM) VII. Similarly, TESs can also be generated in the β configuration.

Subsequently, by using the COMSOL software, the simulated eigenmodes of the SSH model composed of 6 defect cavities are shown in Figure 2(f), representing the magnitude of the electric field. The defect cavities within the units exhibit diagonal mode coupling, while the defect cavities between units exhibit lateral mode coupling, consistent with the Hamiltonian in Eq. (1). In this case, the lattice constant *a* is set at 600 nm, the diameter of the regular PC cavities is 0.9*a, and the diameter of the defect cavities is 0.3*a. Figure 2(f) shows that at the resonant frequency of 200.5 THz, the α configuration exhibits localized TESs of the diagonal mode, while at the resonant frequency of 201.5 THz, the β configuration also displays localized TESs of the diagonal mode. This phenomenon indicates that in our defect-cavity PCs, it is not necessary to change the inter-unit spacing to tune coupling strength, constructing an SSH model based on differences in mode coupling strength is equally effective. Additionally, the robustness of this SSH model is also verified. By randomly perturbing the positions of the defect cavities, and randomly missing the cavitied in PCs, noticing that the operations will nearly not break the chiral symmetry of SSH model, the TESs remain present. More details can refer to the SM II. The above results are all simulated in 2D with effective refractive index of silicon (*n*_si_ = 2.8) by using the commercial software COMSOL Multiphysics.

## 4 Transmissible TESs in SSH model

**Figure 3:**
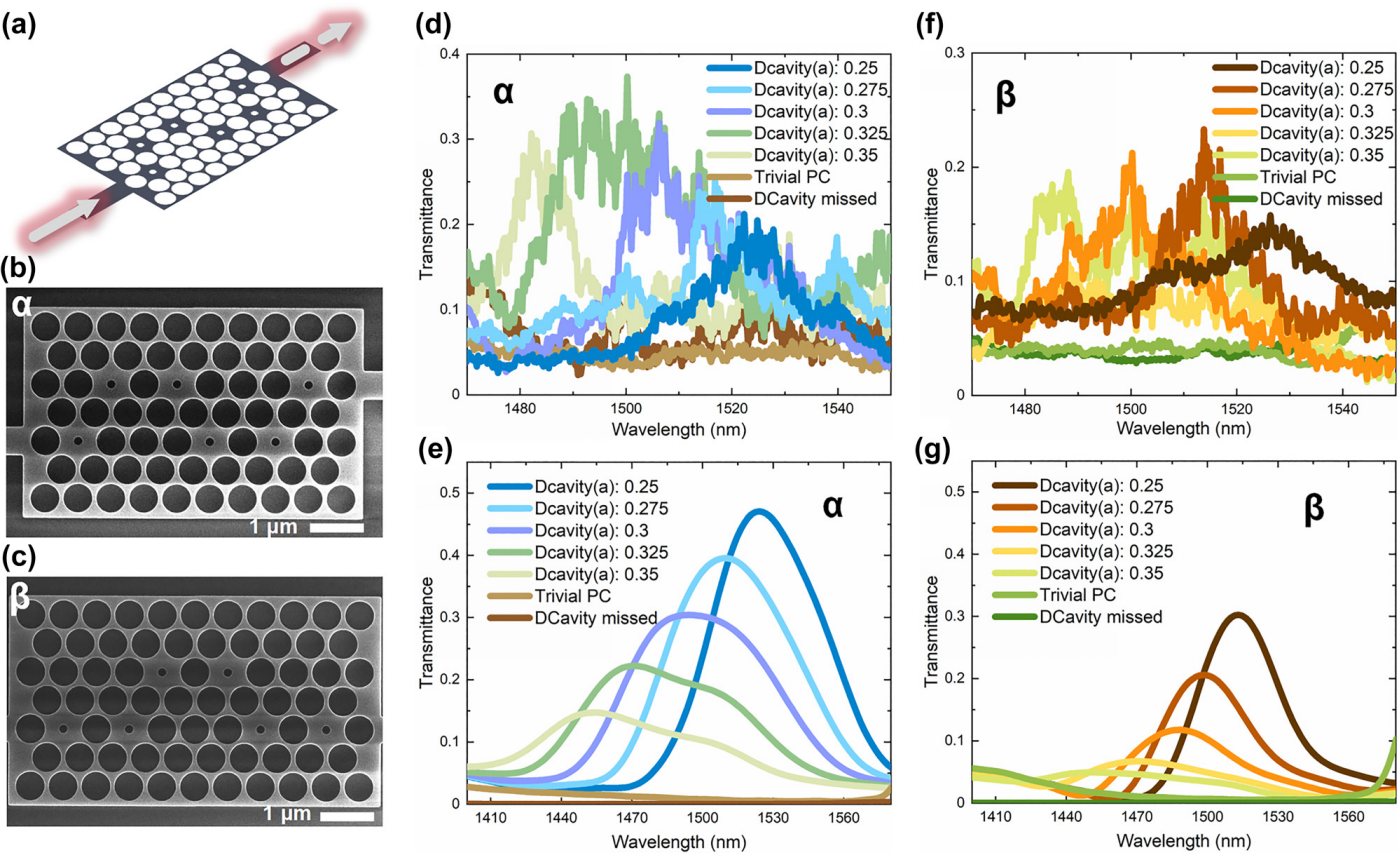
Experimental and simulated results for transmissible TESs in SSH model. (a) Illustration of α configuration with waveguide segments added at both ends of the structure to create transmissible TESs. (b) SEM image of the α configuration that can support transmissible TESs. (c) SEM image of the β configuration that can support transmissible TESs. (d) Experimental spectra for the α configurations with varying-diameter defect cavities. The control-group structures, including the trivial PCs and PCs without the varied-diameter holes, are also measured. (e) Simulated spectra for the α configurations with varying-diameter defect cavities, and the control groups. (f) Experimental spectra for the β configurations with varying-diameter defect cavities, and the control groups. (g) Simulated spectra for the β configurations with varying-diameter defect cavities, and the control groups.

Both the α and β configurations can realize the TESs based on the SSH model, commonly found as the localized optical states. However, considering the case of TE modes in our PCs, it is possible to achieve the transmissible TESs through the coupling between defect cavities and boundary waveguides. The transmission of the boundary electric field distribution can occur through tunneling, enabling ultrafast modulation speeds. Note that the tunneling effects can only facilitate the transmission of TESs when the device size is small, otherwise, the decaying tails cannot sense what happens at the other boundary. More details can refer to SM VI. Figure 3(a) illustrates the use of the α configuration, with waveguide segments added at both ends of the structure to excite and measure transmissible TESs. This allows for on-chip excitation and signal reception. Taking the α configuration as an example, with regular air hole diameters of 0.9*a and defect cavity diameters of 0.3*a, simulations are performed to calculate the transmission spectrum of the structure in the excitation wavelength range from 1400 nm to 1580 nm. The expanded range of excitation wavelengths in the simulations, from 1000 nm to 1800 nm, are illustrated in SM VI, showing the wavelength ranges of the guiding bands and the bandgap. It is evident that the TESs are located within the band gap. The excitation from the left waveguide leads to the transmission spectrum obtained from the right waveguide, the spectrum is shown by the purple line in Figure 3(e). A transmission peak appears around a wavelength of 1500 nm, and this wavelength falls within the photonic bandgap created by the trivial PC composed of air holes with diameter of 0.9*a. In trivial PCs, all light would be blocked, but this transmission peak in defect-cavity PCs is a result of the introduction of defect cavities and thus generating TESs. Observing the energy eigenvalues represented by the blue line in Figure 1(b), it is clear that the resonance wavelength of the defect cavities shifts towards shorter wavelengths as the defect cavity diameter increases. Therefore, a series of on-chip SSH configurations is designed with defect cavity of different diameters, starting from the diameter of 0.25*a and increasing in 0.025*a increments up to the diameter of 0.35*a. The transmission spectra for these configurations are simulated, shown in Figure 3(e), indicating that the transmissible TESs correspond to wavelengths that shift towards shorter wavelengths as the defect cavity diameter increases. For comparison, similar calculations are performed for trivial PCs and PCs without the varied-diameter holes. Their transmission spectra are represented by the brown and tan lines in Figure 3(e). In the wavelength range where TESs exist, both the two PC structures, as control groups, exhibit almost zero transmittance. The simulated transmittances on PCs with defect cavities are normalized using the transmittance under excitation in the guiding band of trivial PCs.

Furthermore, we fabricated samples based on SOI (silicon-on-insulator) substrate by using electron beam lithography and inductively coupled plasma etching technique. The top silicon layer had a thickness of 220 nm. Figure 3(b) provides the scanning electron microscope (SEM) image of the α configuration that can support transmissible TESs. The scale bar in the figure represents a size of 1 μm. In this configuration, the lattice constant was 600 nm, the diameter of the regular cavities was 540 nm, and the diameter of the defect cavities varied in accordance with (0.25, Δ0.025, 0.35) *a. The measurement system was based on a fiber-coupled setup. Supercontinuum laser (YSL SC-5) is coupled into a single mode fiber, and then coupled from the fiber to the chip through etched grating. After passing through the sample, the optical signal on the chip is coupled to the fiber through grating on the other side, and then introduced into the spectrometer (Andor SR 303i-b). The experimental results for the α configuration are shown in Figure 3(d). The transmission peak positions, which were 1523 nm (defect cavities with diameter of 0.25*a), 1516 nm (defect cavities with diameter of 0.275*a), 1500 nm (defect cavities with diameter of 0.3*a), 1489 nm (defect cavities with diameter of 0.325*a), and 1483 nm (defect cavities with diameter of 0.35*a), respectively, shifted towards shorter wavelengths with increasing defect cavity diameter. This trend aligns with the simulated results. Noting the presence of small peaks caused by oscillation in the experimental transmission spectra, the positions of the transmission peaks are determined by identifying the spectral line that conforms to a Gaussian profile and has the maximum transmittance. The experimental transmittances were also normalized using the transmittance under excitation in the guiding band of the trivial PCs. It is worth noting that the peak transmission values in the simulation gradually decrease as the defect cavity diameter increases. This is normal because a larger defect cavity diameter means a reduction in the coupling strength of the split modes, leading to a decrease in the strength of the TESs, details can refer to SM III. In contrast, for the experimental results, the peak transmission values gradually increased as the defect cavity diameter increased. This is due to the fact that the coupling grating is not broadband but follows a Gaussian distribution, with its coupling efficiency peak centered around 1500 nm, seen in SM IV. As the wavelength moves away from this central peak, the coupling efficiency gradually decreases.

Similarly, we conducted a series of simulation calculations and experimental measurements for the β configuration. Figure 3(c) shows the SEM image of the β configuration, while Figure 3(g) displays the transmission spectra for varying defect-cavity diameters in the β configuration, along with control groups of trivial PCs and PCs without the varied-diameter holes. All β configurations were subjected to spectral measurements, and the experimental results are depicted in Figure 3(f). The peak positions of the transmission spectra shifted towards shorter wavelengths with increasing defect cavity diameter, consistent with the simulation results. Noting that the transmission intensity of transmissible TESs in the β configuration is generally lower than that in the α configuration. This is because the β configuration employs lateral-coupling units, where the difference in coupling strength between the intra-unit diagonal mode and the inter-unit lateral mode is small, resulting in the intensity reduction in TESs, seen in SM III. As a result, to further enhance the transmission peak of transmissible TESs, increasing the diameter of the regular cavities or reducing the diameter of the defect cavities in PCs are both effective approaches. However, due to fabrication constraints, it is not possible to infinitely widen this difference. In the design of our work, the minimum defect-cavity diameter size was 0.25*a, i.e., 150 nm, and the linewidth between two regular PC cavities was 0.1*a, i.e., 60 nm, all within the current mature range of nanostructure fabrication capabilities. The simulation results here were obtained in a 3D environment, which is consistent with the experimental conditions.

## 5 Wavelength division multiplexer

**Figure 4:**
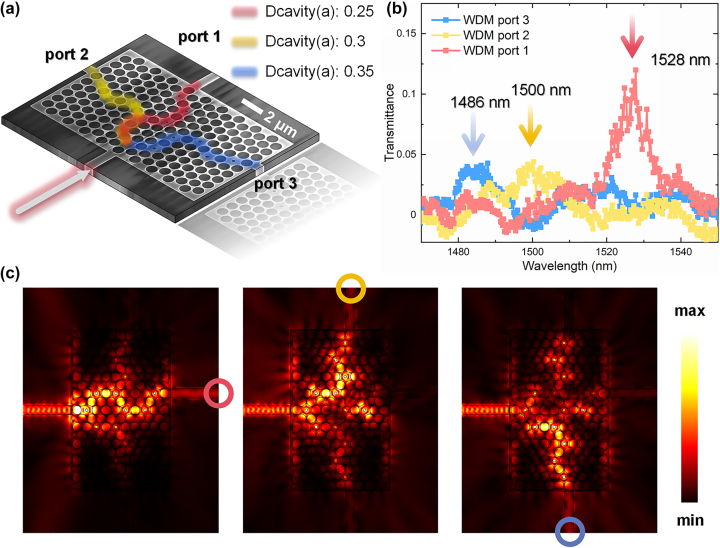
Wavelength division multiplexer. (a) SEM image of the WDM device and paths schematic representation. The three output ports are labeled as port 1 (middle path), port 2 (top path), and port 3 (bottom path), represented by red, yellow, and blue lines, respectively, denoting different propagation paths than can support TESs excited by different wavelengths. Path 1 consists of defect cavities with diameter of 0.25*a. Path 2 consists of defect cavities with diameter of 0.3*a. Path 3 consists of defect cavities with diameter of 0.35*a. All the paths share the first input defect cavity at the left boundary. (b) Experimental transmission spectra for WDM device. Port 1 corresponds to a transmission peak at 1528 nm, port 2 at 1500 nm, and port 3 at 1486 nm. (c) Simulated transmissible TESs mode distributions in WDM device at the three different wavelengths. The red circle on the output indicates port 1, the yellow circle on the output indicates port 2, and the blue circle on the output indicates port 3.

Up to this point, we have successfully implemented the generation of transmissible TESs in 2D PCs with defect cavities based on the 1D SSH topological model. The defect cavity size is a tunable parameter, allowing control of light transmission through the PCs at different wavelengths. This opens up new possibilities for the design of optical micro-nano devices and can be used as a WDM device. Figure 4(a) displays an SEM image of the WDM device and paths schematic representation, consisting of one input port and three output ports with topological PC defect cavities. The three output ports are labeled as port 1 (middle path, α configuration), port 2 (top path, β configuration), and port 3 (bottom path, β configuration), represented by red, yellow, and blue lines, respectively, denoting that different propagation paths that can support TESs excited by different wavelengths. Since there is only one input port, and the three paths share a common defect cavity when entering the PCs, it is important to ensure that the first defect cavity’s size is compatible with the wavelengths corresponding to the three paths to prevent crosstalk.

Based on the above analysis, we designed the WDM with the following defect cavity diameters for each path: Path 1 uses defect cavities with diameter of 0.25*a, Path 2 uses defect cavities with diameter of 0.3*a, and Path 3 uses defect cavities with diameter of 0.35*a. The final experimental transmission spectra are shown in Figure 4(b). Port 1 corresponds to a transmission peak at 1528 nm, port 2 at 1500 nm, and port 3 at 1486 nm. The simulated mode distributions at the three different wavelengths are shown in Figure 4(c). Due to the tunneling effect between the boundaries of the TESs, the internal defect cavities also exhibit electric field distributions with certain intensities. It is obvious that the three paths have distinct transmission peak positions, achieving on-chip WDM functionality. Although port 2 and port 3 exhibit lower transmission intensities due to the β configuration and the sharing defect cavity in the input position, this WDM setup demonstrates that the defect-cavity PC configurations that support on-chip transmissible TESs can be readily processed on a large scale using standard fabrication techniques. Moreover, due to the compact structural design (horizontal length of 6 μm), which inherently ensures a response time of approximately 20 fs for light transmission, and this transmissible TESs leverage the tunneling effect between boundary electric fields, allowing for a response time shorter than the light transmission time, i.e., the response time for a single operation is less than 20 fs. This ease of integration makes the WDM a practical choice for on-chip applications. Furthermore, ongoing development in tunable defect cavity PCs will enhance the prospects for photonic integration in the future.

## 6 Conclusions

We have developed on-chip transmissible TESs utilizing SSH defect cavities on a SOI slab. To create the SSH model, we employed different coupling strengths between the lateral and diagonal modes within the defect-cavity PCs. Two distinct photonic SSH-cavity configurations, referred to as α and β configurations, were analyzed to illustrate the existence of TESs. Taking advantages of the TE mode’s on-chip transmission capability within PCs, we used a waveguide to excite a boundary defect cavity. Intriguingly, we observed that the transmissible light, corresponding to the TESs, could be captured in another boundary defect cavity through waveguide. Moreover, the transmission peak of TESs undergoes a blue shift as the defect cavity size increases. Consequently, by tuning the size of the SSH defect cavity, we successfully demonstrated on-chip WDM device in experimental settings, which the ultrafast response time for one operation is less than 20 fs.

This work breaks away from the conventional notion of 0D topological edge states being localized and combines the transmission properties of 2D defect-cavity photonic crystal waveguides. As a result, it achieves on-chip, transmissible 0D topological edge states. By controlling the defect cavity size, it also creates an integrated, tunable, and ultrafast on-chip topological optical device with WDM capabilities. This comprehensive on-chip device provides a feasible solution for advancing the applications of topological photonics, taking it beyond fundamental research and opening doors to future topological optical devices such as in optical communication, optical storage, optical computing, and topological quantum computing.

## Supplementary Material

Supplementary Material Details
