# Supplementary material for: Transmissible topological edge states based on Su–Schrieffer–Heeger photonic crystals with defect cavities
Source: Nanophotonics. 2024 Jan 23;13(8):1397–406. doi: 10.1515/nanoph-2023-0744 (PMC11636502; doi:10.1515/nanoph-2023-0744)
Supplement: Supplementary file 1 — Supplementary Material Details [file j_nanoph-2023-0744_suppl_001.pdf]

Supplementary Materials

Transmissible Topological Edge States Based on Su-Schrieffer-Heeger Photonic Crystals with Defect Cavities

Qiuchen Yan[1,†,], Rui Ma[1,†], Chong Li[1], Qinghong Lyu[1], Xiaoyong Hu[1,2,3,4,‡], and Qihuang Gong[1,2,3,4]*

Contents

I. Eigenvalue distributions of varied-diameter air hole in trivial PCs

II. Robustness of defect-cavity SSH model

III. Influence of coupling differences on TESs

IV. The bandwidth of grating

V. Supplemental SEM images

VI. Implementation of transmissible TESs

VII. Odd and even symmetry modes of TESs

I. Eigenvalue distributions of varied-diameter air hole in trivial PCs

Figure SM1 shows the eigenvalue distributions of varied-diameter air hole in trivial PCs. The varied diameter of air hole in trivial PCs is from 0.1*a to 0.5*a, and there are no any available modes within the bandgap, which is different from the defect cavities.

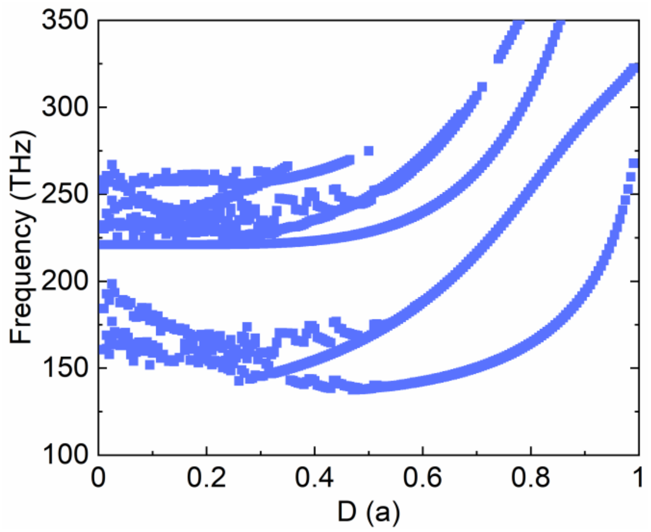

Figure SM1. Eigenvalue distributions of varied-diameter air hole in trivial PCs.

II. Robustness of defect-cavity SSH model

We first simulated the changes in the eigenmodes of the defect cavity in PCs after introducing arbitrary position perturbations in a single defect cavity, as shown in Figure SM2. We found that when the total position perturbations were no more than 20 nm, i.e., $\Delta x + \Delta y \leq 20$ nm, the corresponding wavelengths of the eigenmodes did not undergo significant shifts. This ensures that the chiral symmetry of the SSH model will not be broken even after introducing some positional perturbations. In addition, we also calculated the mode spectra for the case of dual-cavity arrangements. Take example of the case on diagonal coupling arrangement, the corresponding wavelengths of the eigenmodes do not change significantly when adding the position perturbations, as depicted in Figure SM3. The phenomenon is the same in the case of lateral coupling arrangement. In other words, the coupling strength between different modes remains nearly unchanged when adding the position perturbations. Moreover, the TESs

distributions of α configuration, where the random positional perturbations and missing cavities are added, are demonstrated in Figure SM4. We observed that random positional perturbations had minimal impact on the resonant frequencies and mode distribution of TESs, but the absence of certain cavities resulted in the degenerate TESs becoming non-degenerate. Figure SM5 similarly presents the resonant frequencies and mode distribution of TESs for the β configuration with random positional perturbations and missing cavities.

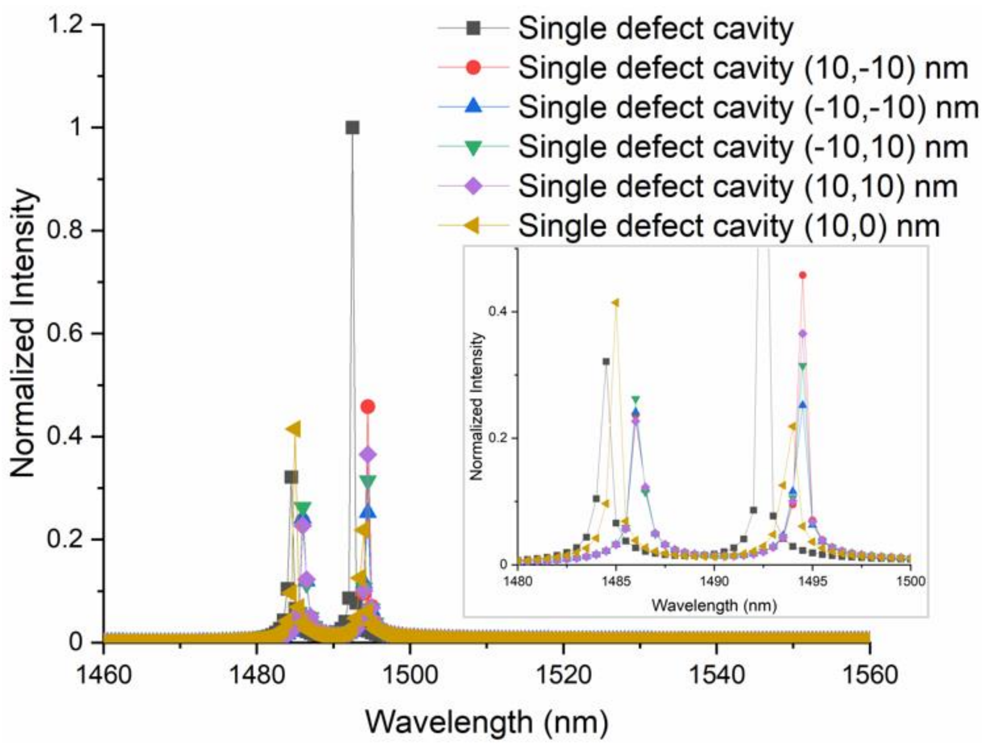

Figure SM2. Mode spectra in a unit PC after introducing arbitrary position perturbations to a single defect cavity. Notice that (10, -10) nm in the figure means the position shift of the single defect cavity along x direction is 10 nm, and the position shift along y direction is -10 nm. The inset is the enlarged part of the mode spectra.

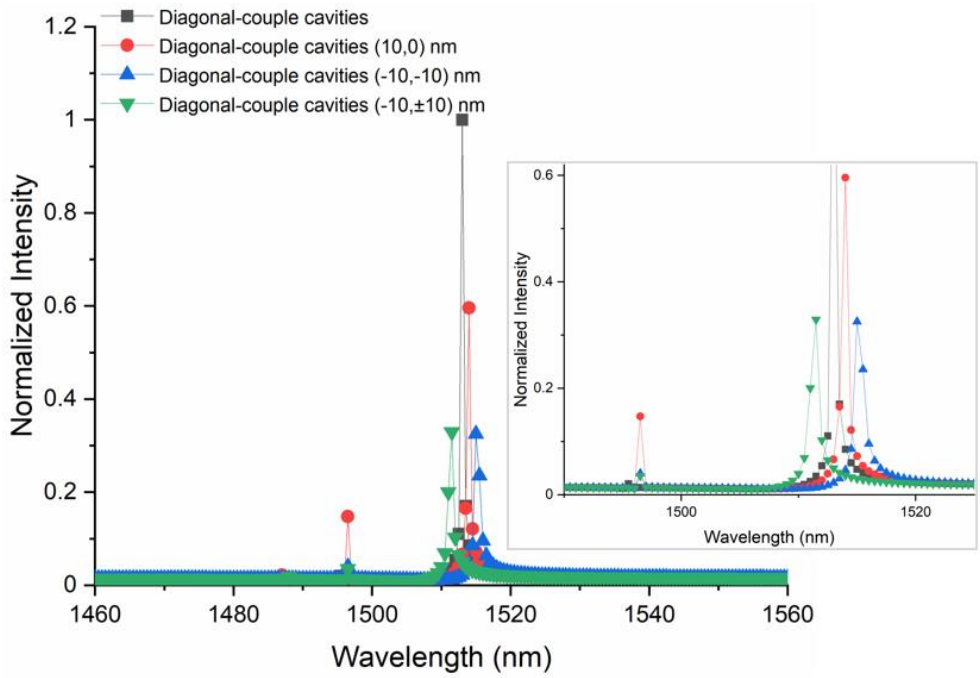

Figure SM3. Mode spectra in a unit PC after introducing arbitrary position perturbations to diagonal-coupling arrangement dual cavity. Notice that (10, 0) nm in the figure means the position shift of the two defect cavities are the same, i.e., the position shift along x direction is 10 nm, and the position shift along y direction is 0 nm. However, (-10, ±10) nm in the figure means the position shift of the two defect cavities are different, i.e., for the left defect cavity, the position shift along x direction is -10 nm, and the position shift along y direction is 10 nm; for the right defect cavity, the position shift along x direction is -10 nm, and the position shift along y direction is -10 nm. The inset is the enlarged part of the mode spectra.

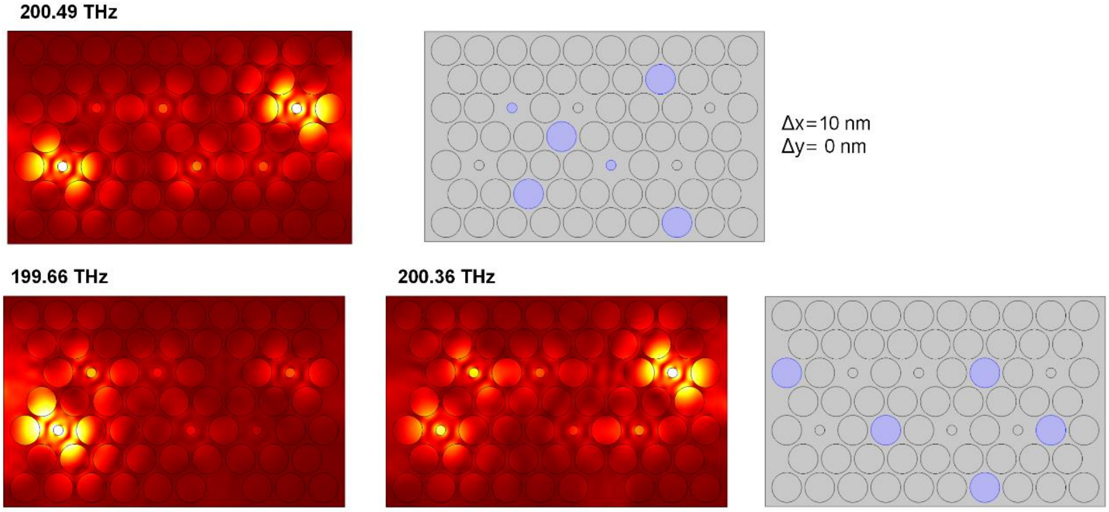

Figure SM4. The resonant frequencies and mode distribution of TESs for α configuration. The top-line figures are the case of adding random positional perturbations, and the bottom-line figures are the case of adding missing-cavity perturbations.

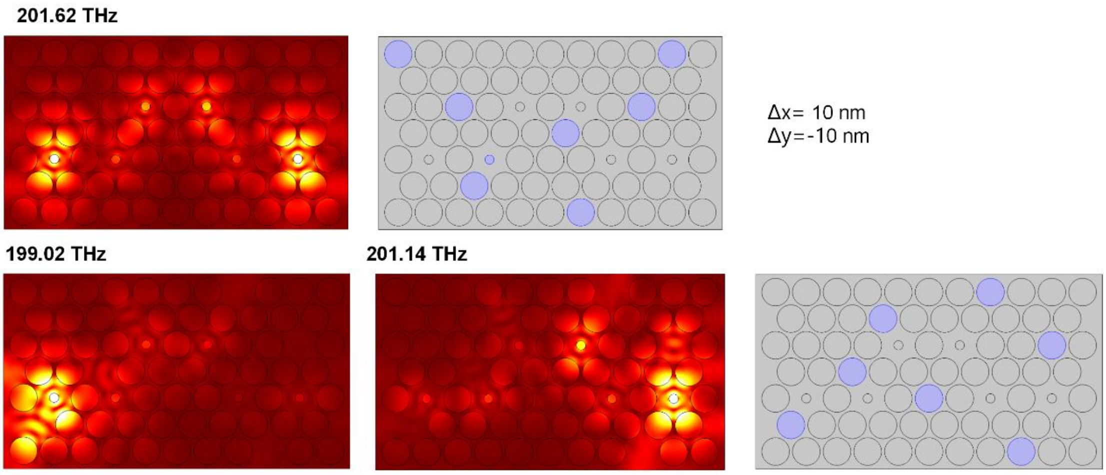

Figure SM5. The resonant frequencies and mode distribution of TESs for β configuration. The top-line figures are the case of adding random positional perturbations, and the bottom-line figures are the case of adding missing-cavity perturbations.

III. Influence of coupling differences on TESs

From the analysis in the main text, we can qualitatively determine the mode coupling strengths in the lateral coupling arrangement and diagonal coupling arrangement based on the degree of mode splitting, i.e.,

$$\kappa_{diagonal\ mode}^{diagonal\ arrangement} = 1.5$$

$$\kappa_{lateral\ mode}^{diagonal\ arrangement} = 2.7$$

$$\kappa_{diagonal\ mode}^{lateral\ arrangement} = 1.2$$

$$\kappa_{lateral\ mode}^{lateral\ arrangement} = 4.1$$

In SSH model, for the α configuration, the Hamiltonian can be written as follows:

$$\hat{H} = \kappa_{diagonal\ mode}^{diagonal\ arrangement} \sum_{m=1}^{n} (|m, B >< m, A| + h.c.)$$

$$+ \kappa_{lateral\ mode}^{lateral\ arrangement} \sum_{m=1}^{n-1} (|m+1, A >< m, B| + h.c.).$$

While for β configuration, the Hamiltonian can be written as follows:

$$\hat{H} = \kappa_{diagonal\ mode}^{lateral\ arrangement} \sum_{m=1}^{n} (|m, B >< m, A| + h.c.)$$

$$+ \kappa_{lateral\ mode}^{diagonal\ arrangement} \sum_{m=1}^{n-1} (|m+1, A >< m, B| + h.c.).$$

Notice that the TESs will be obvious when there is a significant difference in coupling strengths between intra-unit and inter-unit interactions. The significant difference in coupling strengths ensures that the isolated solutions, corresponding to TESs, in the bandgap are more difficult to transition into the bulk band. In α configuration, the coupling difference is $\frac{\kappa_{lateral\ mode}^{lateral\ arrangement}}{\kappa_{diagonal\ mode}^{diagonal\ arrangement}} = 2.733$, and in β configuration, the coupling difference is $\frac{\kappa_{lateral\ mode}^{diagonal\ arrangement}}{\kappa_{diagonal\ mode}^{lateral\ arrangement}} = 2.25$, therefore, the TESs in α configurations will be more obvious than TESs in β configurations. Similarly, the TESs will be more obvious in the case of PCs with smaller defect cavities.

IV. The bandwidth of grating

We used the etched grating to coupling light from the normal to the plane. The period size of the grating is 600 nm, and the etched width for each cycle is 230 nm. The etch depth is 60 nm. Figure SM6 shows the simulated transmittance results for grating. The peak of coupling efficiency is around 1500 nm. Figure SM7 is the SEM image for the fabricated grating.

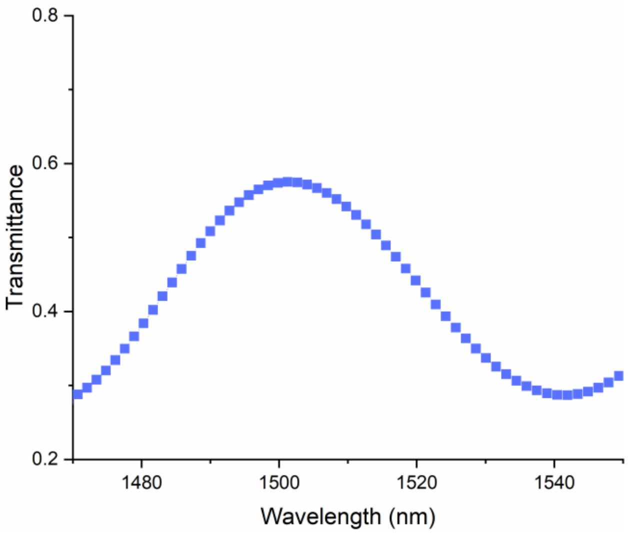

Figure SM6. Simulated transmittance results for grating.

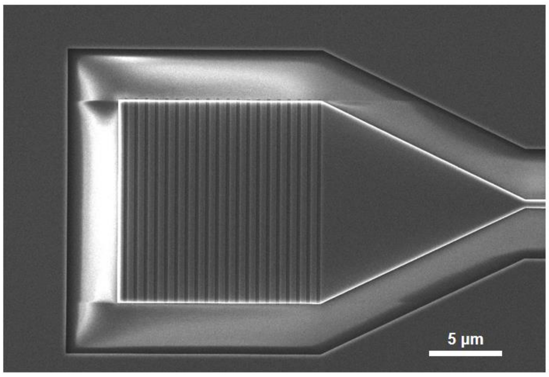

Figure SM7. SEM image for the fabricated grating.

V. Supplemental SEM images

Here, we supply other SEM images, including the trivial PCs and PCs without defect cavity in both α configurations and β configurations in Figure SM8.

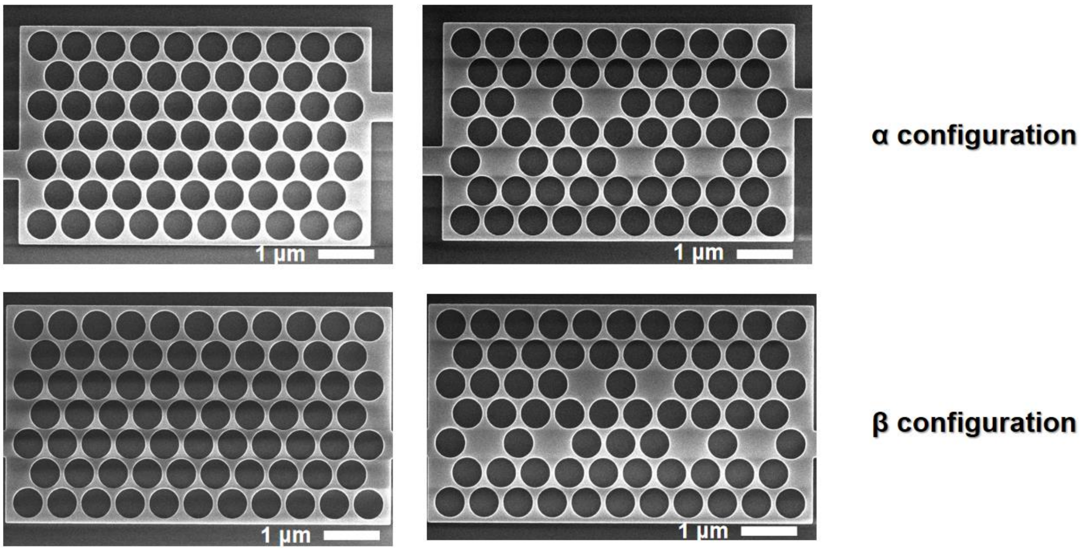

Figure SM8. SEM images of the trivial PCs and PCs without the varied-diameter holes in both α configurations and β configurations.

VI. Implementation of transmissible TESs

Under periodic boundary conditions, it can determine the topological properties of SSH system by calculating the winding number. While the observation of topological edge states occurs under open boundary conditions, with a fixed number of lattices in the SSH model. For example, as the Figure 2e in the main text shows, the quasi-energy band diagram of the eigenvalue distribution is constructed with 100 particles, and the two eigenvalues in the band gap correspond to the distribution of eigenmodes of the topological edge states, as shown in Figure SM9a. It can be observed that there is no electric field distribution inside the SSH structure at this point. In contrast, under the case of the bulk mode, as shown in Figure SM9b, there is an electric field distribution inside the SSH structure. In the context of this manuscript, within the defect cavity configuration of the SSH photonic crystal, a total of 6 defect cavities are included. The energy eigenvalue distribution and the modes of topological edge states under this condition can be calculated. As shown in Figure SM9c-d, modes 3 and 4 are located in the band gap, corresponding to the topological edge states. By comparing Figure SM9a and SM9d, it can be observed that in Figure SM9d, the internal structure of the topological edge states still exhibits electric field distributions. This is the reason in this

study that allows the generation of transmissible edge states. The tunneling from one boundary to another involves the overlap of the electric fields in the intermediate defect cavity. The transmission of the boundary electric field distribution can occur through tunneling, enabling ultrafast modulation speeds.

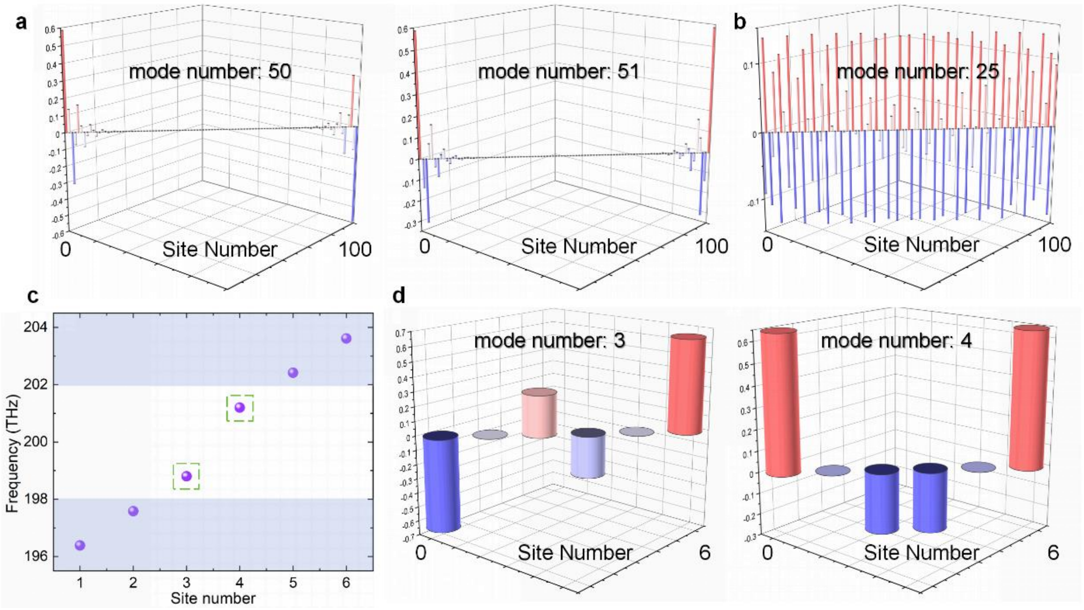

Figure SM9. The topological edge state mode distributions and bulk mode distributions of different SSH structures. a. Topological edge state mode distribution with anti-symmetry (mode number of 50) and topological edge state mode distribution with symmetry (mode number of 51) in the SSH chains with 100 particles. b. Bulk mode distribution in the SSH chains with 100 particles. c. The energy eigenvalue distributions of SSH chains with 6 particles, corresponding to both the α configuration and β configuration in the main text. d. Topological edge state mode distribution with anti-symmetry (mode number of 3) and topological edge state mode distribution with symmetry (mode number of 4) in the SSH chains with 6 particles.

We also expanded the range of excitation wavelengths in the simulations, from 1000 nm to 1800 nm, as illustrated in Figure SM10. The transmission spectrum of the regions I and III shows the wavelength range of the guiding bands. For instance, around 1700 nm at longer wavelengths, the transmission peak position remains unchanged regardless of variations in the defect cavity diameter. This contrasts sharply with the phenomenon observed in the topological edge states within the region II (the band gap), where the transmission peak position shifts to longer wavelengths as the defect cavity size decreases.

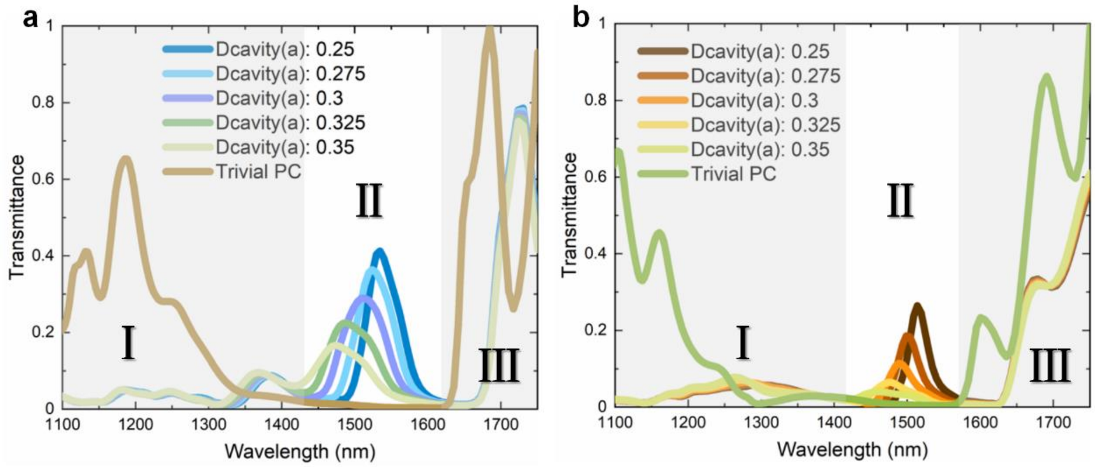

Figure SM10. Simulated transmission spectra of defect-cavity SSH configurations and trivial photonic crystal under the expanded range of excitation wavelengths from 1000 nm to 1800 nm, which cover three regions. The region II is the band gap of the trivial photonic crystals. a. The α configurations. b. The β configurations.

VII. Odd and even symmetry modes of TESs

Figure 2e illustrates two topological edge states in the bandgap corresponding to odd-symmetric and even-symmetric mode distributions, as shown in Figure SM9. Regardless of whether the number of cavities is 100 or 6, there are always two topological edge states in the band gap. In terms of the mode distributions for specific configurations, there are also odd-symmetric and even-symmetric mode distributions, as depicted in Figure SM11. Here, we present the mode distributions of the Ex (x component of the electric field), making it clear to distinguish between odd-symmetric and even-symmetric modes. In the main text, we chose the stronger mode intensity for the α configuration at the eigenfrequency of 200.5 THz and the topological edge state for the β configuration at the eigenfrequency of 201.5 THz.

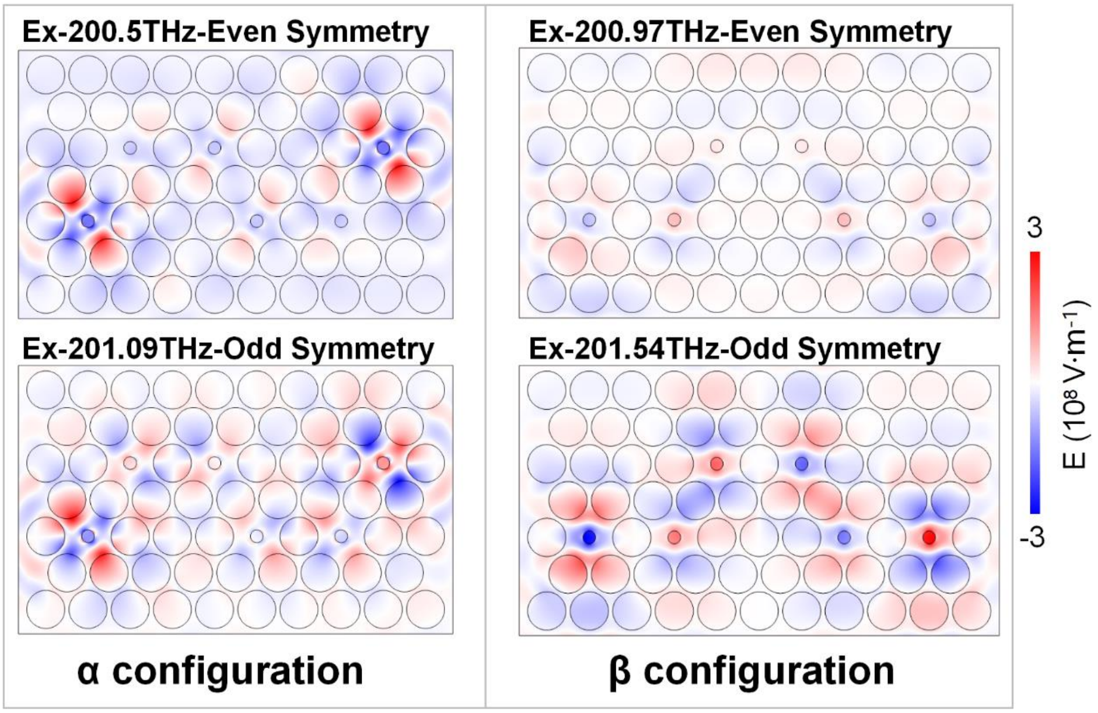

Figure SM11. The x-direction electric field component of topological edge states with odd symmetry modes (in the top row of the figure) and even symmetry modes (in the bottom row of the figure) of α configuration (left panel of the figure) and β configuration (right panel of the figure).
